# Age-Related Changes in Pre- and Postsynaptic Partners of the Cholinergic C-Boutons in Wild-Type and SOD1^G93A^ Lumbar Motoneurons

**DOI:** 10.1371/journal.pone.0135525

**Published:** 2015-08-25

**Authors:** Léa Milan, Gilles Courtand, Laura Cardoit, Frédérique Masmejean, Grégory Barrière, Jean-René Cazalets, Maurice Garret, Sandrine S. Bertrand

**Affiliations:** INCIA, Université de Bordeaux, CNRS UMR5287, Bordeaux, France; Inserm, FRANCE

## Abstract

Large cholinergic synaptic terminals known as C-boutons densely innervate the soma and proximal dendrites of motoneurons that are prone to neurodegeneration in amyotrophic lateral sclerosis (ALS). Studies using the Cu/Zn-superoxide dismutase (SOD1) mouse model of ALS have generated conflicting data regarding C-bouton alterations exhibited during ALS pathogenesis. In the present work, a longitudinal study combining immunohistochemistry, biochemical approaches and extra- and intra-cellular electrophysiological recordings revealed that the whole spinal cholinergic system is modified in the SOD1 mouse model of ALS compared to wild type (WT) mice as early as the second postnatal week. In WT motoneurons, both C-bouton terminals and associated M2 postsynaptic receptors presented a complex age-related dynamic that appeared completely disrupted in SOD1 motoneurons. Indeed, parallel to C-bouton morphological alterations, analysis of confocal images revealed a clustering process of M2 receptors during WT motoneuron development and maturation that was absent in SOD1 motoneurons. Our data demonstrated for the first time that the lamina X cholinergic interneurons, the neuronal source of C-boutons, are over-abundant in high lumbar segments in SOD1 mice and are subject to neurodegeneration in the SOD1 animal model. Finally, we showed that early C-bouton system alterations have no physiological impact on the cholinergic neuromodulation of newborn motoneurons. Altogether, these data suggest a complete reconfiguration of the spinal cholinergic system in SOD1 spinal networks that could be part of the compensatory mechanisms established during spinal development.

## Introduction

Synapses converging onto the soma and the highly branched dendritic tree of motoneurons (Mns) have been divided into five different types: S, T, M, F and C [1–4]. C-terminals or C-boutons are characterized by spherical synaptic vesicles and the presence of postsynaptic subsynaptic cisterns [5]. These synapses are cholinergic [6–8] and originate from interneurons located around the central canal that express the transcription factor Dbx-1, a maker of the V0 interneuron cohort [9,10].

Given the importance of cholinergic neuromodulation in spinal locomotor networks, numerous studies have investigated C-bouton morphology in physiological and pathological conditions [11,12]. These synapses are extremely plastic and exhibit morphological changes after operant conditioning [13] or spinal hemisection [14]. Recently, the postsynaptic membrane (PSM) adjacent to C-boutons on Mns has been described as a highly organized structure with interdigitating clusters of type-2 muscarinic receptors (M2), small conductance calcium-activated potassium channels (SK2/3) and Kv2.1 channels [12].

Amyotrophic lateral sclerosis (ALS) is a neurodegenerative disorder that affects spinal motoneurons (Mns) and cortical motor neurons. In ALS patients and rodent models of the disease, it has been shown that Mns innervating fast-twitch muscles (fast Mns) are much more vulnerable to neurodegenerative processes than those connecting slow-twitch muscle fibers (slow Mns) [15,16]. Interestingly, C-boutons are noticeably more prevalent on fast Mns compared to slow Mns, and groups of Mns that are preserved in ALS, such as the ocular motor nuclei, do not present C-boutons [7]. C-terminals thus appear to be a hallmark of Mns prone to cell death in ALS. In the Cu/Zn-superoxide dismutase (SOD1) mouse model of ALS, the timeline of morphological changes of C-boutons during ALS pathogenesis has been investigated [17–20]. Although these studies all demonstrate early alterations of C-boutons in presymptomatic stages of the disease, they report conflicting data. These studies also raise the unexplored question of whether C-bouton alterations originate solely from their postsynaptic targets, the Mns, or whether impairments of the lamina X cholinergic interneurons, are also involved.

In the present work, by combining immunohistochemistry, extra- and intracellular electrophysiology and biochemistry, we revealed major reorganizations of the spinal cholinergic system in SOD1 mice compared to wild-type (WT) animals as early as the second postnatal week. Our data identified that (1) C-bouton evolution exhibits different temporal profiles in WT and SOD1 Mns, (2) M2 receptors present a complex dynamic in C-bouton PSM in WT Mns that is completely disrupted in SOD1 Mns, (3) lamina X cholinergic interneurons are more abundant in high lumbar segments in SOD1 mice compared to WT, (4) these neurons are subjected to neurodegeneration in the SOD1 ALS model and (5) early C-bouton alterations have no physiological impact on cholinergic neuromodulation of newborn Mns.

## Materials and Methods

### Animals and ethics

This study was carried out in strict accordance with the recommendations of the European Committee Council Directive. The protocol was approved by the local ethics committee of the University of Bordeaux (permit number 5012031A). High-expressor hemizygous G93A SOD1 male mice (strain B6SLJ-Tg-(SOD1^G93A^)1Gur/J from The Jackson Laboratory, Bar Habor, ME, USA) were bred with non-transgenic B6SJLF1/J females (Janvier Laboratory, France). Male offspring expressing the mutant SOD1-G93A gene (hereafter referred to as SOD1 mice) and age-matched male WT littermates were used. Mice were genotyped from genomic DNA purified from tail biopsies by PCR using the following primers: 5’ CATCAGCCCTAATCCATCTGA 3’ (forward), 5’ CGCGACTAACAATCAAAGTGA 3’ (reverse). All of the experiments and analyses presented here were performed blind to the genotype of the animals.

### Immunohistochemistry

For double immunofluorescence labeling, WT and SOD1 mice aged 1 postnatal day (P1), P10, P21, P40, P75 and P100 (Table 1) were anesthetized with an intraperitoneal injection of 20% urethane. After the loss of nociceptive reflexes, mice were perfused with cold phosphate-buffered saline (PBS), followed by cold 4% paraformaldehyde (PAF) in PBS. Spinal cords were dissected free, post-fixed in 4% PAF for 2 hours, and then cryoprotected overnight in 20% PBS-sucrose. Lumbar spinal cords were cryosectioned in transverse slices (30 μm thick) and mounted on slides. Sections were incubated overnight in the primary antibody mixture containing 0.3% triton and 1% bovine serum albumin (BSA). After washing in PBS, sections were incubated for 1 h with secondary antibodies in 0.3% triton and 1% BSA. The following primary antibodies were used: goat anti-CholineAcetylTransferase (ChAT; 1:100, Millipore AB144P); rat anti-M2 receptor (1:400, Millipore MAB367) and rabbit anti-cleaved caspase 3 (1:50, Cell Signaling 9661). All of the secondary antibodies, Alexa fluor 568 donkey anti-goat, Alexa fluor 488 donkey anti-rat and Alexa fluor 488 donkey anti-rabbit (Invitrogen), were used at 1:500 dilution.

**Table 1:** Number of animals used and motoneurons (MN) analyzed in each age group in wild-type (WT) and SOD1 mice.

	P1		P10		P21		P40		P75		P100	
	WT	SOD1	WT	SOD1	WT	SOD1	WT	SOD1	WT	SOD1	WT	SOD1
Animal number	4	4	6	6	7	6	8	6	6	7	5	8
MN Number	100	83	130	133	148	131	130	126	124	145	116	156

For the cholinergic interneuron counting experiments, a biotinylated anti-goat (RPN1025V, Amersham) secondary antibody was used, and ChAT-positive neurons were visualized with successive incubations with streptavidin (Vectastain ABC kit, Biovalley), 3,3’-diaminobenzidine (DAB) and hydrogen peroxide solutions.

### Acquisition and Image analysis

Images were acquired on a Leica SP5 confocal microscope using a 63x oil immersion lens and analyzed with FIJI (ImageJ) plugins developed at the laboratory. For each animal, three different slides of lumbar spinal cord sections were randomly selected and processed for immunohistochemistry. Images of three randomly sampled lumbar spinal cord sections were then acquired for each slide. Table 1 summarizes the number of animals used and motoneurons analyzed in each age group and animal genotype. Care was taken not to image motoneurons with vacuolar structures. Images of Mn soma composed of 15 slices in a stack (0.25 μm step) were acquired. Mns were manually selected and outlined on the maximum image intensity obtained by maximum pixels intensity projection along the z-axis of the ChAT channel. From this selection, values of area, perimeter and geometric center (centroid) of Mns were calculated. C-boutons were automatically detected according to defined morphologic criteria and pixel intensity in a region of interest (ROI) defined by the expansion (2 μm) of the Mn contour defined above.

To analyze M2 receptor expression in the PSM adjacent to C-boutons, a sum projection along the z-axis was performed on both the ChAT and M2 channels. Constant-width bands (2.83 μm) of both channels centered on the Mn contour were extracted from this projection. Variations in the intensity of these two bands were measured along their length with the FIJI plot profile tool. ROIs containing C-boutons in the ChAT channel were automatically detected and applied to the M2 channel after an expansion of 1 μm. M2 labeling area and intensity under C-boutons were then computed. The M2 intensity under C-bouton was normalized by the mean M2 intensity computed in the Mn membrane devoid of C-boutons.

### Slide scanning and interneuron counting

ChAT/DAB immunostained slices from P10, P40 and P100 WT and SOD1 mice were scanned with a Nanozoomer (Nanozoomer 2.0 HT Hamamatsu C9600-12). Lamina X cholinergic interneurons located in an area 80 μm around the central canal were manually counted in individual slices with the NDP viewer software (Hamamatsu). To count neurons in a similar spinal region in each age-matched animal group, the intermediolateral neurons (IML) were used as anatomical landmarks. IML are cholinergic neurons, thereby appearing in ChAT/DAB labeling, which express a well-defined anatomical distribution throughout spinal segments. Indeed, IML extend from the first thoracic segment (T1) through to the second lumbar segment (L2). The measured lumbar segment lengths were 450, 950 and 1000 μm in P10, P40 and P100 mice, respectively. The individual slice thickness was 30 μm, and cholinergic lamina X interneurons were counted in 15 slices at P10 and 33 slices at P40-P100 more rostral from the last slice containing IML and in 50 slices at P10 and 100 slices at P40-P100 more caudal from the last slice containing IML. With this method, cholinergic lamina X neurons could be accurately counted from the L2 to the L5 cord segments.

### Western blot analysis

M2 muscarinic receptor protein expression was assessed in spinal cord tissue from at least 4 P1, P10, P21, P40, P75 and P100 SOD1 mice and 4 WT littermates using a previously described protocol [21]. Briefly, proteins extracted from tissue samples were separated on 10% SDS-polyacrylamide gels and blotted onto nitrocellulose membranes. The primary antibody binding (rat anti-M2 receptor, 1:300, Millipore MAB367) was probed with an anti-rat horseradish peroxidase-conjugated secondary antibody (1:5000, Jackson) and visualized with an enhanced chemiluminescence kit (Lumiglo/Eurobio). An antibody against β-actin (1:2000, Synaptic System) was used to control for equal protein loading. Bands were quantified using ImageJ, and the results obtained for each sample were normalized to the amount of β-actin measured.

### Extracellular recordings in *in vitro* spinal cord preparations from newborn mice

11 WT and 8 SOD1 mice aged P1-P3 were anesthetized using isoflurane until reflexes were lost and were decapitated. The spinal cord was then dissected free and placed ventral side up in a recording chamber. All dissections and recording procedures were performed under continuous perfusion with artificial cerebrospinal fluid (aCSF) containing the following (in mM): NaCl 130, KCl 3, CaCl_2_ 2.5, MgSO_4_ 1.3, NaH_2_PO_4_ 0.58, NaHCO_3_ 25 and glucose 10, with a pH of 7.4 when bubbled with 95% O_2_ + 5% CO_2_ at room temperature (24–26°C). Motor outputs were recorded extracellularly from the lumbar ventral roots using glass suction electrodes. In each *in vitro* spinal cord preparation, motor outputs from the right and left lumbar 2 (rL2, lL2, respectively) and one L5 ventral root were simultaneously recorded to investigate both the bilateral segmental alternation and the flexor/extensor activity [22,23]. Neurograms were amplified, displayed and stored using a classical electrophysiological device. Motor burst parameters were computed using custom-made Matlab-based software. For each preparation, the burst amplitude values were normalized to the amplitude measured in the presence of an agonist of the N- methyl-D-L-aspartate (NMDA) receptors, NMA and serotonin (5-HT) (16 μM each) prior to the addition of oxotremorine to the bath.

### Whole-cell recordings in spinal cord slices

In 5 deeply anesthetized P7-P11 WT and 12 SOD1 mice, a laminectomy was performed to remove the spinal cord in an ice-cold sucrose-based saline solution containing the following: 2 mM KCl, 0.5 mM CaCl_2_, 7 mM MgCl_2_, 1.15 mM NaH_2_PO_4_, 26 mM NaHCO_3_, 11 mM glucose, and 205 mM sucrose. The saline was bubbled with 95% O_2_, 5% CO_2_. Transverse slices (350 μm) of the spinal lumbar enlargement were cut with a vibroslicer and then transferred in a holding chamber. Slices were allowed to recover in oxygenated aCSF (130 mM NaCl, 3 mM KCl, 2.5 mM CaCl_2_, 1.3 mM MgSO_4_, 0.58 mM NaH_2_PO_4_, 25 mM NaHCO_3_, 10 mM glucose, 1 mM sodium pyruvate and 5 μM (reduced) L-glutathione) for at least 1 hour at 30°C. The slices were transferred to a second holding chamber containing the same aCSF at 30°C but without sodium pyruvate and (reduced) L-glutathione before recordings. Whole-cell current-clamp recordings from Mns, identified by their relatively large size in lamina IX, were made under visual control with a Multiclamp 700B amplifier. Recording glass microelectrodes (4–7 MΩ) were filled with the following: 120 mM K-gluconate, 20 mM KCl, 0.1 mM MgCl_2_, 1 mM EGTA, 10 mM HEPES, 0.1 mM CaCl_2_, 0.1 mM GTP, 0.2 mM cAMP, 0.1 mM leupeptin, 77 mM d-mannitol, and 3 mM Na_2_-ATP, with a pH of 7.3. A bipolar tungsten electrode was used to stimulate axons crossing through the ventral commissure. The polysynaptic transmission was decreased using a high cation-containing aCSF with 7.5 mM CaCl_2_ and 8 mM MgSO_4_ in order to record monosynaptic excitatory postsynaptic potentials (EPSPs). Strychnine (1 μM), Gabazine (1 μM) and DNQX (5 μM) were added throughout the experiments to block glycinergic, GABA_A_ and AMPA/kainate receptor activation, respectively. Oxotremorine (500 μM) was bath applied on the slices. All of the experiments were performed at room temperature (25°C).

### Statistical analysis

Statistical analyses were performed on raw data. C-bouton morphological parameters were analyzed using SPSS statistics (IBM). The other statistical analyses were conducted using the GraphPad Prism software (GraphPad Prism). Comparisons between two conditions were tested using Student’s *t*-test. Two-way analyses of variance (ANOVAs) with Sidak’s multiple comparison tests were performed to evaluate drug/age and mouse genotype effects. All data are expressed as the means ± SEM. Asterisks in the figures and tables indicate positive significances (p<0.05). For C-bouton parameter analysis (and lamina X cholinergic interneurons counting, Levene’s test was employed to assess equality of variance in each age and genotype groups. In the absence of significant differences, data were pooled across age-matched mice of similar genotype.

## Results

### Age-related morphological changes in C-boutons are different between WT and SOD1 mice

**Fig 1:**
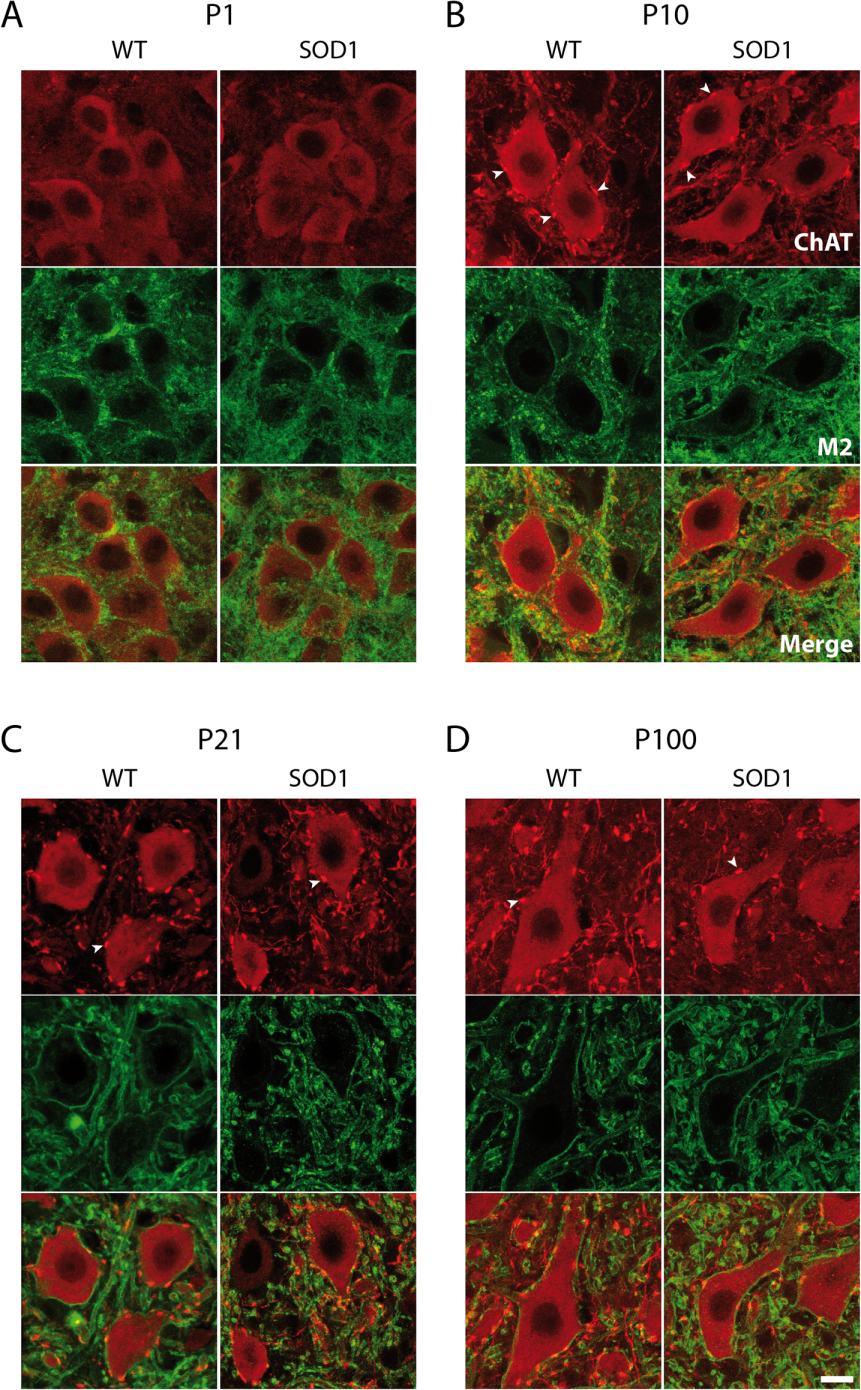
Immunostaining of C-boutons and M2 muscarinic receptors in wild type and SOD1 lumbar motoneurons. Lamina IX confocal microscopy photomicrographs of double-immunofluorescence labeling directed against CholineAcetylTransferase (ChAT, red channel) and M2 muscarinic receptors (green channel) in wild type (WT) and SOD1 postnatal day 1 (P1, **A**), P10 (**B**), P21 (**C**) and P100 (**D**) mice. Arrowheads point to cholinergic large synapses juxtaposed to motoneurons: the C-boutons. Note the absence of C-boutons in P1 motoneurons in these experimental conditions. Scale bar: 15 μm.

**Fig 2:**
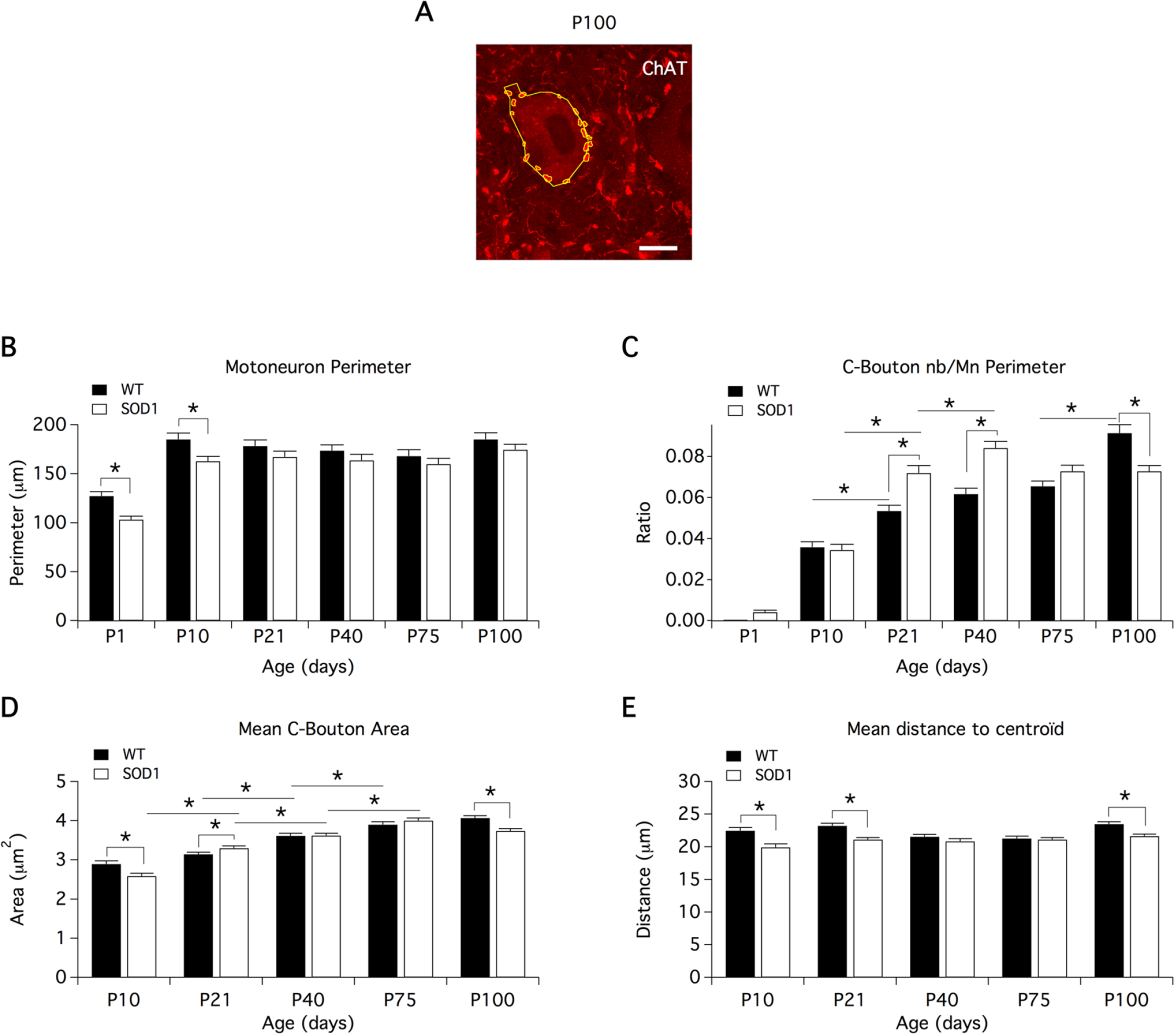
Age-related changes in morphological parameters of lumbar motoneurons and C-boutons in wild type and SOD1 mice. (**A)** Example of automatic detection of C-boutons surrounding a P100 motoneuron in CholineAcetylTransferase (ChAT) labeling images. Scale bar: 15 μm. Histograms of the motoneuron perimeter (**B**), C-bouton number normalized by motoneuron (Mn) perimeter (**C**), mean-C-bouton area (**D**) and mean distance of C-bouton centroid from Mn centroid (**E**) as a function of animal age expressed in postnatal days (P) in wild type (WT, black bars) and SOD1 (white bars) mice. Comparisons between groups were made with two-way ANOVA with Sidak’s multiple comparison tests. Asterisks indicate positive significance (p<0.05). The number of animals used in each group is stated in Table 1. Note differences in the temporal profiles between WT and SOD1 motoneurons in respect to the density and area of C-boutons.

In the first part of this study, we examined the evolution of C-bouton terminals juxtaposed to Mns throughout the life span of WT and SOD1 mice. For this purpose, ChAT-immunolabeling was performed in the lumbar spinal cords from male SOD1 mice and WT littermates at key developmental stages and over the course of the disease, in P1, P10, P21, P40, P75 and P100 animals (Fig 1A–1D; red channel; data not shown for P40 and P75 mice). Regardless of the mouse genotype, we found that, as previously reported [24], C-boutons are absent or too small to be detected in P1 animals in our experimental conditions (Fig 1A) but are easily distinguishable as large ChAT-immunopositive structures surrounding Mn somata and proximal dendrites in P10 mice (arrowheads in Fig 1B). The mean C-bouton number and the mean C-bouton area and position on Mn soma and proximal dendrites were then computed in P10, P21, P40, P75 and P100 WT (Fig 2, black bars) and SOD1 (Fig 2, white bars) mice. As C-bouton number can vary with Mn size, we measured Mn soma perimeter (Fig 2B) and expressed the results as the density of C-boutons on Mns (C-bouton number/Mn perimeter, Fig 2C). This analysis showed, first, that in juvenile stages (P1-P10), the perimeter of lumbar SOD1 Mns were significantly smaller than age-matched WT Mns (Fig 2B). We also observed that in WT animals, the density of C-boutons juxtaposed to Mns increased from P10 to P21, stabilized until P75, and then increased again in P100 mice (Fig 2C), while the mean C-bouton area exhibited a progressive increase with age (Fig 2D). This temporal profile was different in SOD1 mice where C-bouton density strongly increased from P10 to P40 as in WT but decreased at P75 (Fig 2C). In parallel, the mean C-bouton area progressively augmented with age until P75 and decreased at P100 (Fig 2D). Interestingly, pair-wise comparison of data obtained in each age group between WT and SOD1 mice revealed that the density of C-terminals was significantly higher at P21 and P40 in SOD1 mice and significantly lower at P100 compared to WT mice (Fig 2C). In addition, the mean C-bouton area was found to be significantly lower in P10 and P100 and significantly higher in P21 SOD1 mice compared to WT animals (Fig 2D). In a next step, we computed the distance between C-bouton centroids and Mn centroid to see whether the spatial distribution of C-boutons differs between SOD1 and WT MNs and whether C-boutons juxtaposed to SOD1 MNs exhibit a particular topographic pattern of vulnerability during ALS pathogenesis. We observed that this parameter did not evolve with age in either WT or SOD1 mice (Fig 2E). C-boutons were located within a radius of 20–23 μm around the Mn centroid as early as P10. Because Mns were smaller in size in P10 SOD1 mice compared to WT, the C-boutons appeared more clustered in these neurons (Fig 2E). In contrast, although P21 and P100 Mns exhibited similar perimeter values in both genotypes, C-boutons appeared significantly closer to the Mn centroid in SOD1 P21 and P100 animals compared to WT mice.

Altogether, these data show early alterations in the developing pattern of C-boutons juxtaposed to lumbar Mns and a loss of the most distal of these cholinergic synapses in presymptomatic stages (P100) in the SOD1 mice.

### Longitudinal analysis of M2 receptor expression in WT and SOD1 Mns

**Fig 3:**
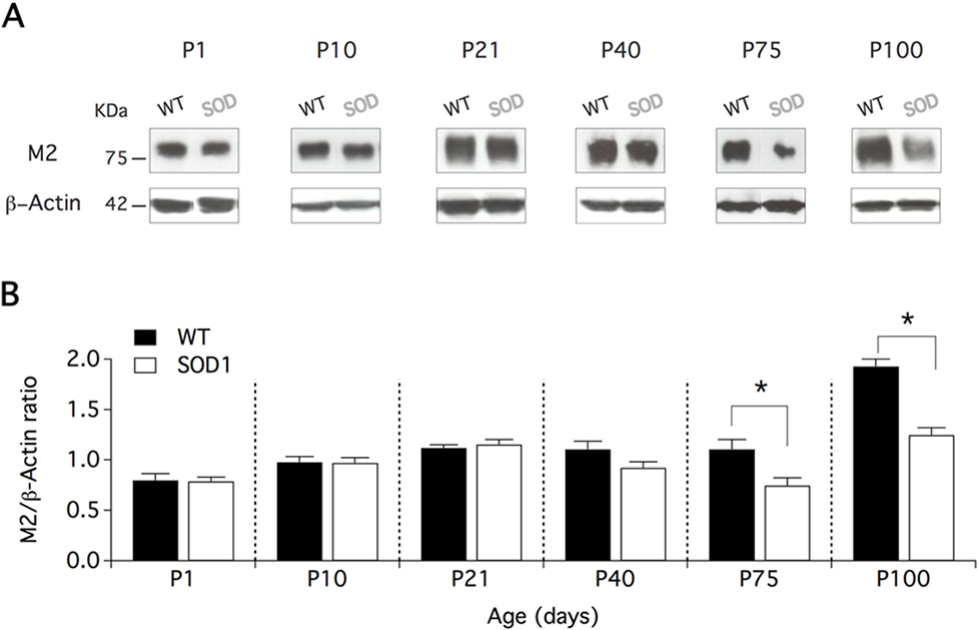
M2 receptor expression is decreased in presymptomatic stages in the SOD1 ventral spinal cord. **(A)** Representative protein levels of the M2 muscarinic receptor detected by western blot in wild type (WT) and SOD1 (SOD) mice at different postnatal (P) stages. All blots were reprobed for β-actin as a loading control. The molecular weight in kDa is shown on the left side of the picture. (**B**) Mean M2 receptor/β-actin expression ratio as a function of animal age in WT (black bars) and SOD1 (white bars) mice. At least four WT and four SOD1 mice were tested in each group. Comparisons between groups were made with two-way ANOVA with Sidak’s multiple comparison tests. Asterisks indicate positive significance (p<0.05).

Anatomical studies have shown accumulations of M2 receptors in the PSM of C-boutons [25] and electrophysiological experiments have further demonstrated the role of these muscarinic receptors in Mn cholinergic modulation [10,26]. As C-boutons exhibit morphological alterations in SOD1 lumbar Mns compared to WT, we addressed the question of whether the postsynaptic partners of these synapses were also modified. For this purpose, a western blot analysis was first performed to compare M2 receptor expression in the ventral spinal cord of WT and SOD1 mice from birth to presymptomatic stages (P100). Fig 3 shows that the M2 protein level was similar in the ventral spinal cord of WT and SOD1 mice from P1 to P21 (Fig 3A and 3B). In P40 animals, the M2 protein content in SOD1 cords tended to decrease compared to WT and became significantly smaller at P75 and P100 (Fig 3A and 3B). These results suggest that the global M2 protein level begins to decrease in presymptomatic stages in the ventral spinal cord of SOD1 mice.

**Fig 4:**
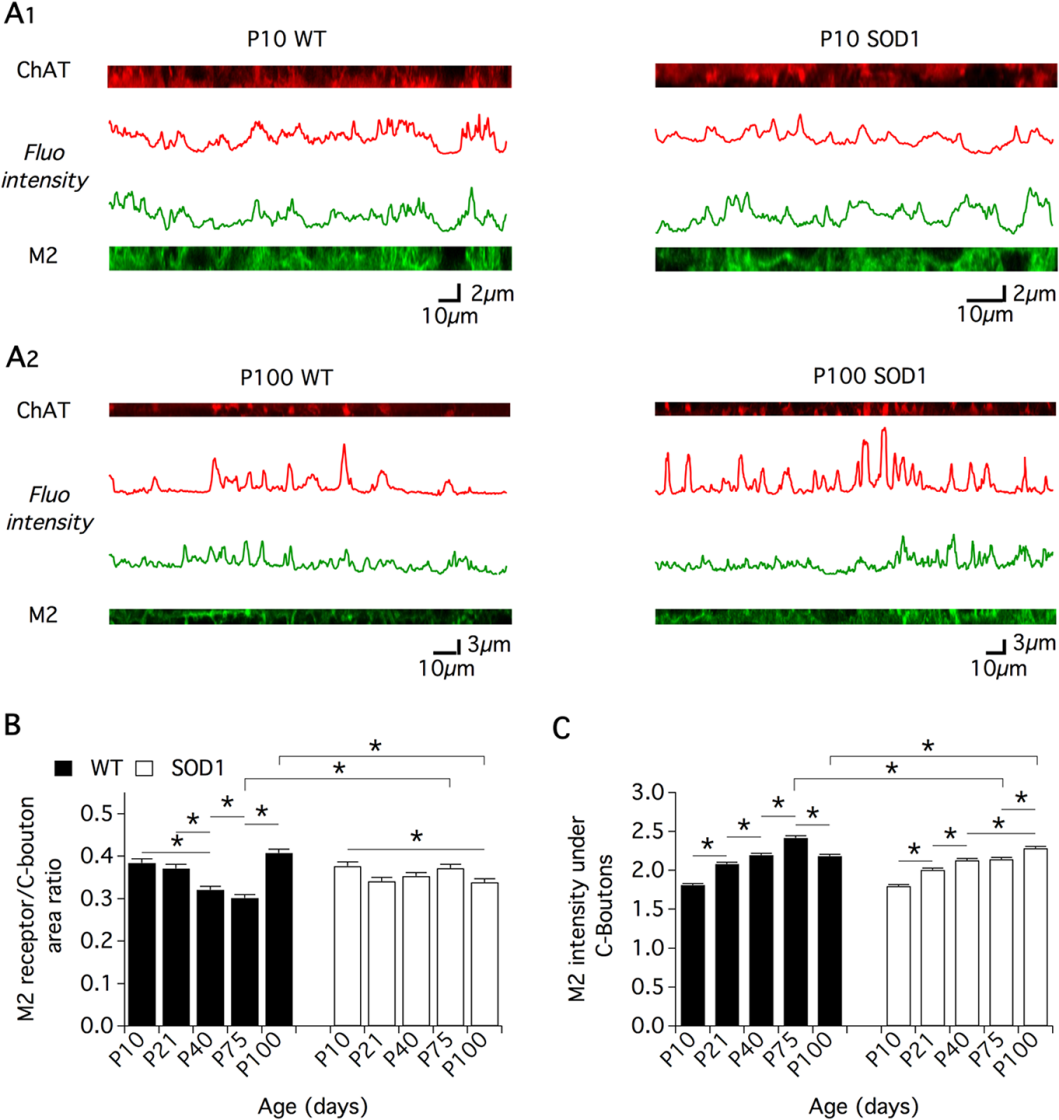
M2 receptor expression in the postsynaptic membrane of C-boutons. (**A)** Representative intensity plot profiles of CholineAcetylTransferase (ChAT, red channel and trace) and M2 receptor (green channel and trace) immunolabeling computed from the contour cutting of wild type (WT, left panels) and SOD1 (right panels) motoneurons of postnatal (P) 10 (**A1**) and P100 (**A2**) mice. (**B)** The portion of postsynaptic membrane containing M2 receptors in front of C-boutons was computed as the ratio of the area of M2 receptor labeling to that of C-bouton labeling and expressed as a function of the postnatal (P) WT (black bars) and SOD1 (white bars) mouse age. (**C)** Histogram of the mean M2 receptor labeling intensity under C-boutons normalized by the mean M2 labeling computed in Mn membrane devoid of C-bouton. Comparisons between groups were made with two-way ANOVA with Sidak’s multiple comparison tests. Asterisks indicate positive significance (p<0.05). The number of animals used in each group is stated in Table 1. Note the significant decrease in the M2 receptor/C-bouton area ratio with age between P10 and P75 in WT motoneurons and the absence of difference in age matched SOD1 motoneurons.

To go one step further, analysis of M2 receptor accumulation under C-boutons during normal development and ALS pathogenesis was performed using ChAT and M2 receptor double immunofluorescence staining (Figs 1 and 4, Table 1). For this purpose, Mn contour cutting including both presynaptic C-boutons in ChAT labeling (red channel in Figs 1 and 4A) and postsynaptic M2 receptors (green channel in Figs 1 and 4A) were realized (for details see Material and Methods). Fig 4 shows the plots of ChAT and M2 labeling intensities of pixels along the Mn contour extracted from a P10 SOD1 Mn (Fig 4A1) and a P100 WT Mn (Fig 4A2) with red peaks highlighting the presence of C-boutons. To characterize M2 receptor expression in C-bouton PSM, two different parameters were computed: 1) the portion of the PSM containing M2 receptors in front of C-boutons expressed as the ratio of the area of M2 receptor labeling to that of C-bouton labeling (Fig 4B) and 2) the M2 receptor labeling intensity under C-boutons normalized by the mean M2 labeling computed in Mn membranes devoid of C-boutons (Fig 4C). The histogram in Fig 4B shows that in WT Mns, the PSM occupied by M2 receptors under C-boutons progressively decreased from P10 to P75 and then re-increased in P100 Mns. This evolution was mirrored in the intensity values of the M2 receptor staining (Fig 4C). Indeed, M2 intensity progressively increased from P10 to P75 and then decreased in P100 Mns. As previously mentioned, we found that the mean C-bouton area exhibits a progressive increase from P10 to P75 in WT Mns (Fig 2D). Our data, therefore, interestingly suggest that in this age range, while M2 receptor expression is enhanced under enlarging C-boutons, these receptors do not spread into the PSM but are sequestrated/clustered in specific areas. At P100, in contrast, we observed a re-increase in the number of C-boutons juxtaposed to lumbar WT Mns (Fig 2C) and a return to immature (P10-like) features with the dispersion of M2 receptors under these synaptic contacts.

Strikingly, this dynamic process is absent in SOD1 Mns. The histograms in Fig 4B show that the ratio between the M2 area and the C-bouton area remained constant from P10 to P75 and decreased at P100 while the M2 intensity progressively increased throughout the time window tested (Fig 4C).

Altogether, these data identify a complex dynamic of M2 receptors in the PSM of C-boutons during development and maturation of Mns that is impaired in SOD1 animals.

### Lamina X cholinergic neurons in WT and SOD1 spinal networks

A growing body of evidence suggests that ALS is not just a motoneuron disease, but that other cellular subtypes are also prone to neurodegeneration in spinal networks during disease progression. C-boutons have been shown to originate from a subclass of V0 interneurons located in lamina X around the central canal [9,10]. In this context, we asked whether the alterations we reported on the pre- and postsynaptic subcellular partners of C-boutons adjacent to SOD1 lumbar Mns could be linked to changes in lamina X cholinergic interneurons (lamina X ChIns).

**Fig 5:**
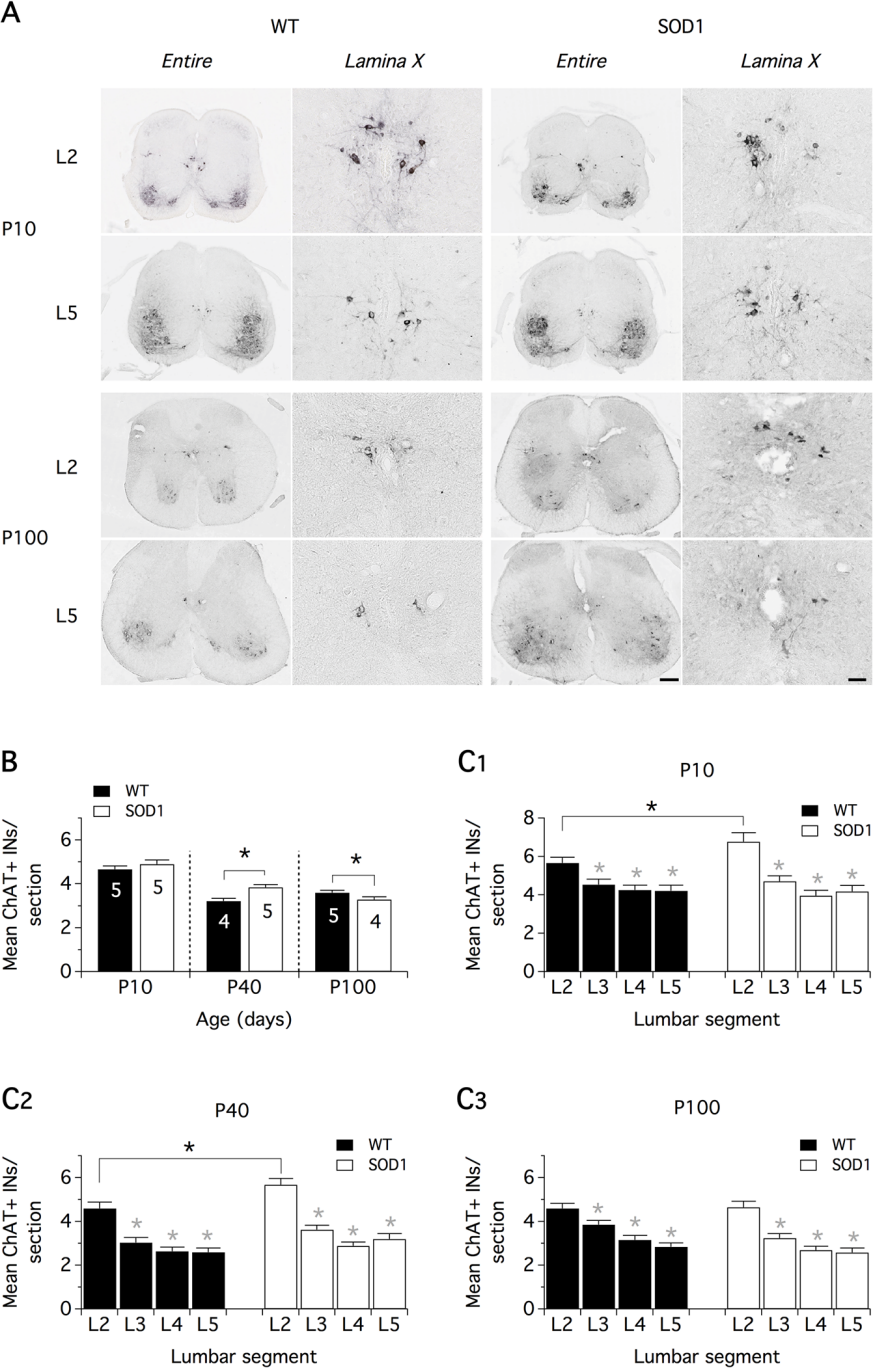
Counting of lamina X cholinergic interneurons in the lumbar spinal cords of wild type and SOD1 mice at different postnatal stages. (**A)** Representative photomicrographs of CholineAcetylTransferase (ChAT) immunopositive neurons in entire spinal cord sections and at a higher magnification in the lamina X of the lumbar 2 (L2) and L5 segments. Images from wild type (WT) and SOD1 mice aged 10 postnatal days (P10, upper panels) and P100 (lower panels) are displayed. Scale bars: 200 μm for entire spinal cord sections and 50 μm for the lamina X magnifications. **(B)** Mean lamina X ChAT-positive interneurons (ChAT+ INs) per spinal cord sections (30 μm thick) in the whole lumbar spinal cord in P10, P40 and P100 WT (black bars) and SOD1 (white bars) mice. (**C)** Mean ChAT+ interneurons (INs) per spinal cord sections for each lumbar segments in P10 (**C1**), P40 (**C2**) and P100 (**C3**) WT (black bars) and SOD1 (white bars) mice. Comparisons between groups were made with two-way ANOVA with Sidak’s multiple comparison tests. Black asterisks indicate positive significance (p<0.05), and the numbers in histogram bars refer to the number of animals tested in each group. Gray asterisks indicate positive significance (p<0.05) between the L2 segments and the other ones in the same group. This analysis revealed an abnormal enrichment of ChAT+ INs in L2 segments of P10 and P40 SOD1 mice and a loss of these neurons in P100 SOD1 animals.

To address this question, in a first series of experiments, lamina X ChIns were visualized using ChAT/DAB staining (Fig 5A) and counted from the L2 to the L5 segments in the spinal cord of five WT and five SOD1 P10, four WT and five SOD1 P40 and five WT and four SOD1 P100 mice (for details see Material and Methods). The data were expressed as the mean number of lamina X ChIns per spinal cord sections (30 μm, Fig 5B and 5C). When computed in the whole lumbar spinal cord, the mean number of DAB positive-neurons per section in the SOD1 spinal cord showed a tendency to be higher at P10 and was significantly increased at P40 compared to WT mice. In contrast, at P100, we observed a significant drop in the mean number of lamina X ChIns per section compared to WT. When the spatial distribution of lamina X ChIns throughout the different lumbar segments was assessed in P10 (Fig 5C1), P40 (Fig 5C2) and P100 (Fig 5C3) mice, we found that, regardless of the animal genotype and age, the L2 segments contained significantly more lamina X ChIns than the other lumbar segments [9]. Interestingly, post-hoc analysis revealed that the mean number of lamina X ChIns per slice was significantly higher in L2 segments in SOD1 P10 and P40 mice compared to WT animals, whereas no significant difference was found between WT and SOD1 L2 segments at P100. This counting approach importantly revealed that SOD1 spinal networks develop with an atypical enrichment of lamina X ChIns in the L2 segments.

**Fig 6:**
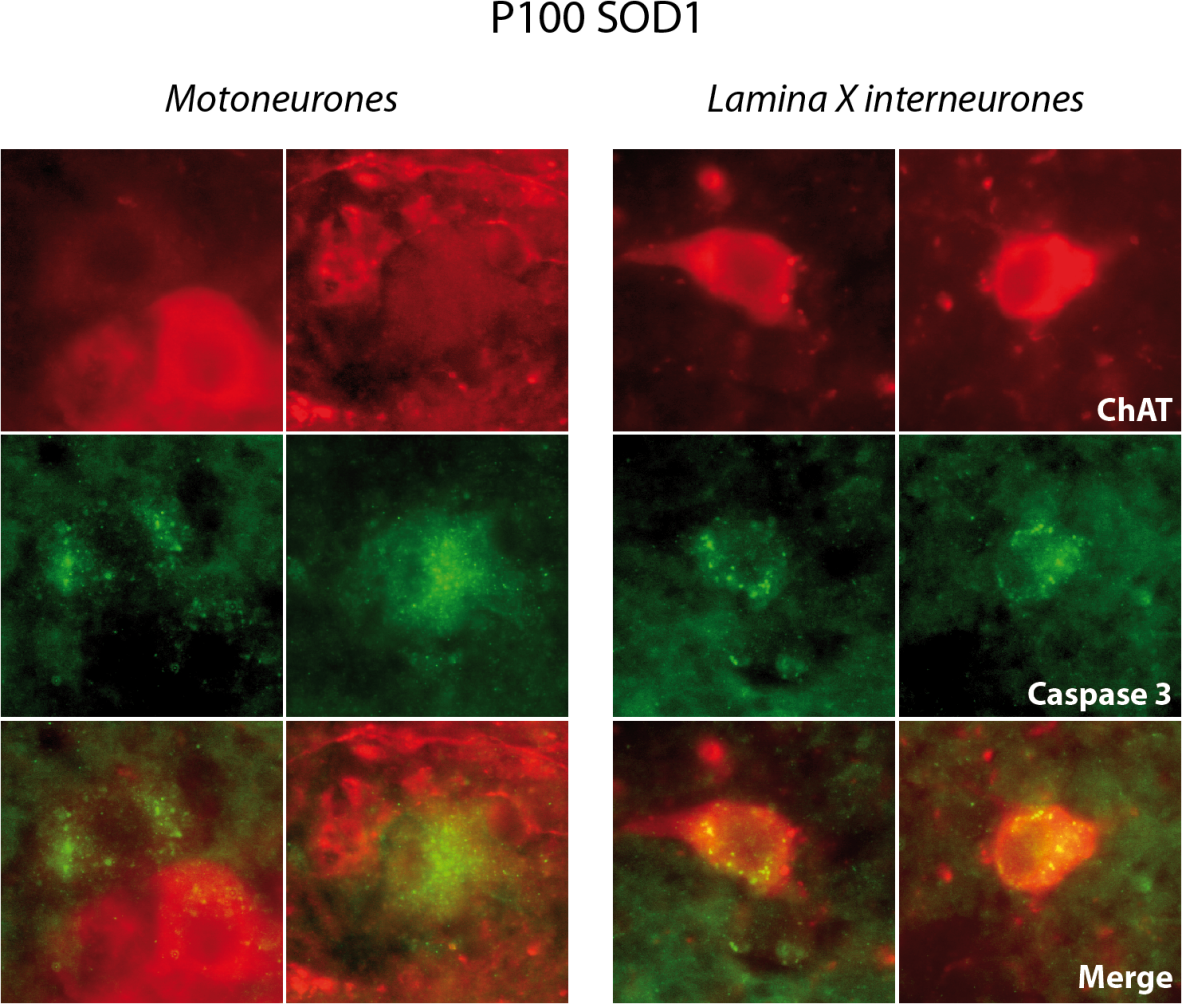
Immunohistochemical observation of cleaved caspase 3 in lamina X cholinergic interneurons in P100 mice. Representative epifluorescence photomicrographs of double labeling of CholineAcetylTransferase (ChAT, red channel) and cleaved caspase 3 (Caspase 3, green channel) in two motoneurons and two lamina X cholinergic interneurons in L2 lumbar segments from 100 postnatal days (P100) SOD1 mice. Note that cleaved caspase 3-positive motoneurons exhibit a weak ChAT staining. Scale bar: 15 μm.

This enrichment was no more present in SOD1 P100 mice, suggesting a loss of lamina X ChIns at this stage. Because the death-mediating protease, caspase-3, is activated in SOD1^G93A^ motoneurons [27,28], we investigated the possible activation of this apoptosis marker in lamina X ChIns in SOD1 P100 spinal cord. While cleaved caspase 3 staining was not detected in L2 spinal cord sections of age-matched WT littermates (data not shown), cleaved caspase 3 immunopositivity was observed both in motoneurons demonstrating weak ChAT expression (Fig 6, left panel) and in lamina X ChIns (Fig 6, right panel) in P100 SOD1 mice. These data further validate that Mns are not the only neurons to degenerate in the SOD1 spinal motor network and indicate for the first time that lamina X ChIns are subject to neurodegeneration in the SOD1 ALS model.

### Extra- and intracellular investigations of the functional impact of early C-bouton alterations

**Fig 7:**
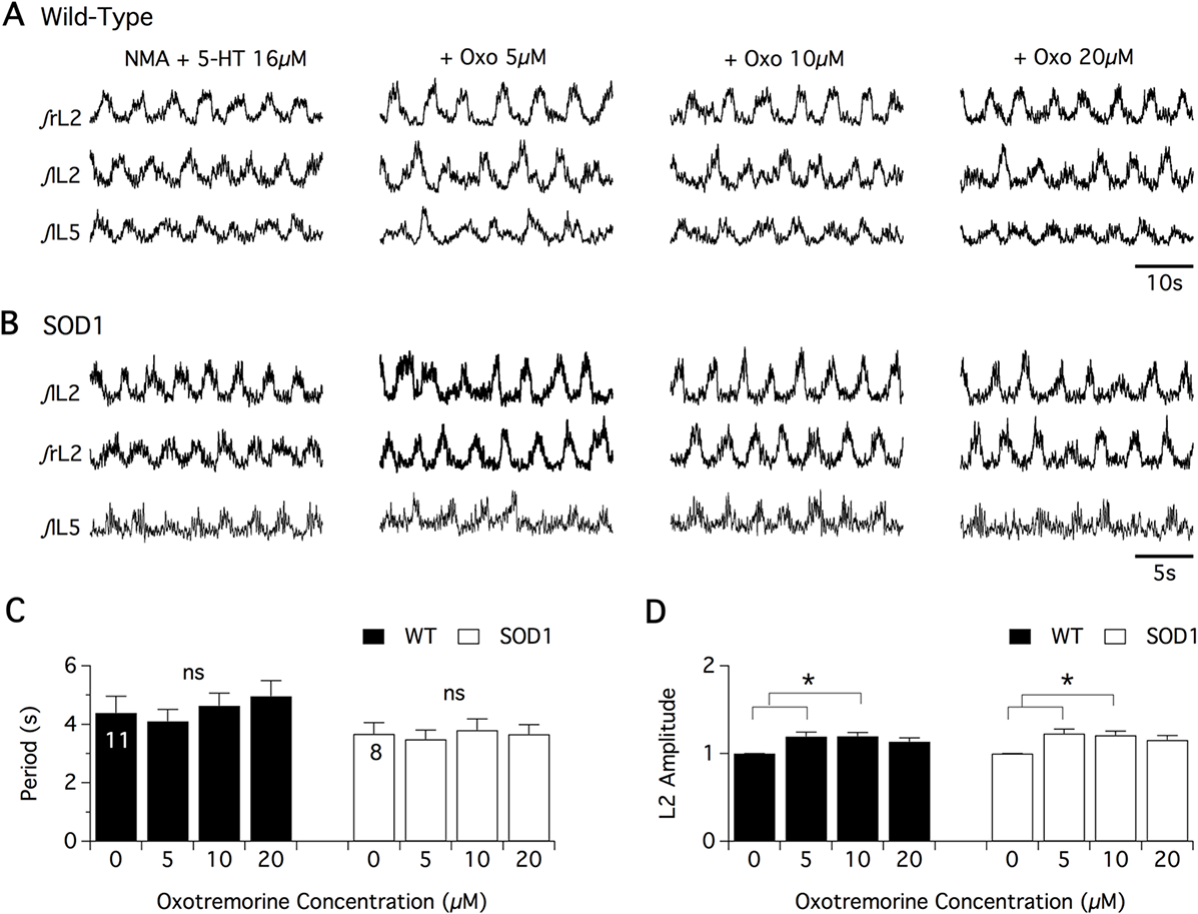
Muscarinic neuromodulatory actions on NMA+5-HT-induced fictive locomotion in SOD1 and age-matched control mice. Representative integrated (ƒ) extracellular recordings from the right, left L2 and left L5 ventral roots (rL2, lL2 and lL5) in the presence of NMA+5-HT (16 μM each) alone or with oxotremorine (Oxo; 5, 10 or 20 μM) in wild type (WT) (**A**) and SOD1 mice (**B**). Plots of the period (**C**) and L2 burst amplitude (**D**) in the absence or presence of oxotremorine bath applied at increasing concentrations on WT (black bars) and SOD1 (white bars) spinal cord preparations. Comparisons between groups were made with two-way ANOVA with Sidak’s multiple comparison tests. Asterisks indicate positive significance levels (p<0.05) and the numbers in histogram bars refer to the number of spinal cord preparations tested.

**Fig 8:**
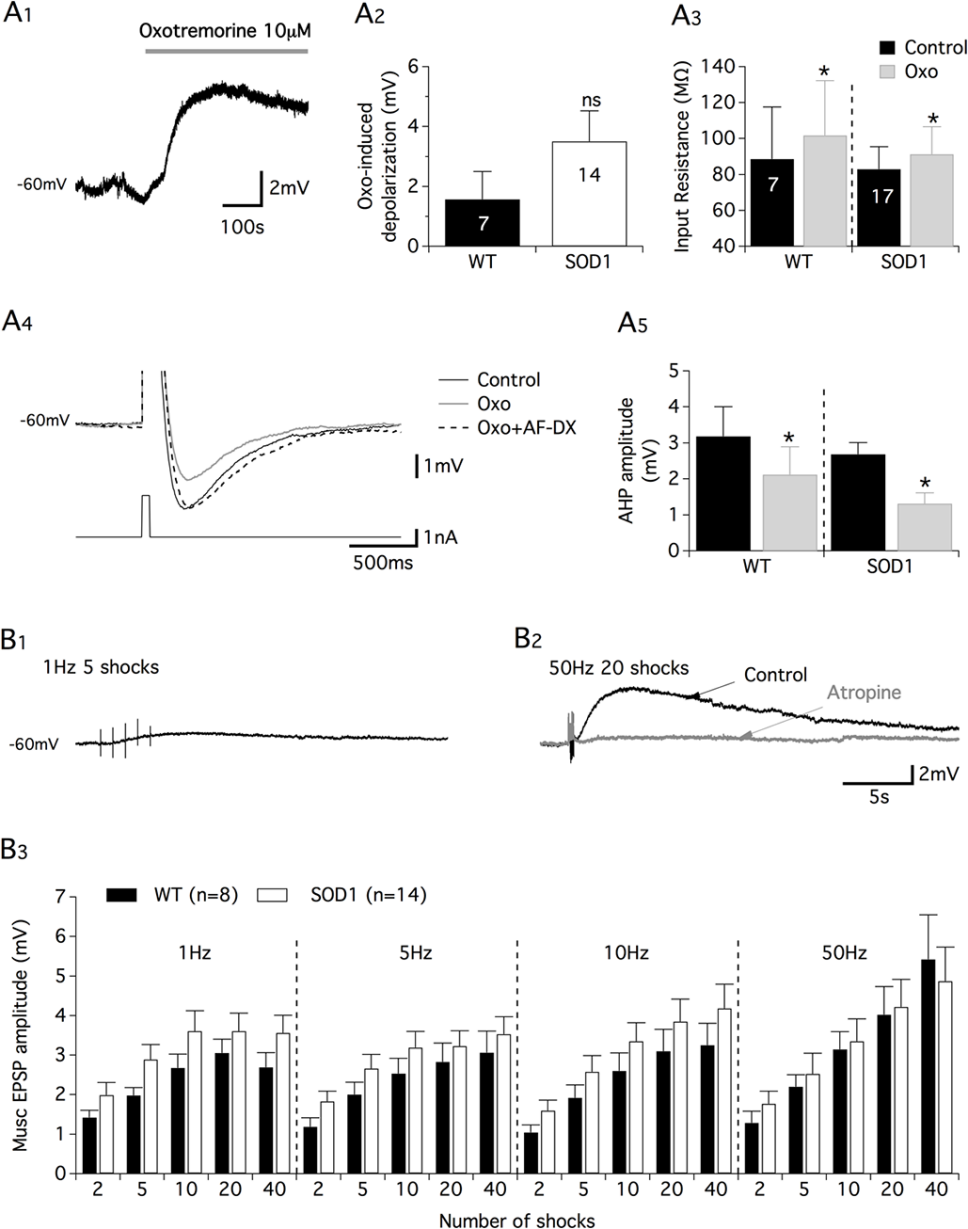
Oxotremorine-induced effects on motoneuron membrane properties and cholinergic commissural transmission in SOD1 and age-matched control mice. **(A)** Representative trace of oxotremorine-induced depolarizations observed in synaptically isolated motoneurons recorded in whole-cell patch-clamp conditions (**A1**). Plots of the mean oxotremorine (oxo)-induced depolarization measured in wild type (WT, black bars) and SOD1 (white bars) motoneurons (**A2**). Comparison was performed with an unpaired Student’s *t*-test (p = 0.24). Plot of the mean membrane input resistance of WT and SOD1 motoneurons in the absence (black bars) and presence (gray bars) of oxotremorine (**A3**). Representative traces of the spike AHP in control (black trace), in the presence of oxotremorine (gray trace) and in the presence of oxotremorine plus the M2 receptor antagonist AF-DX116 (50 μM, dashed trace) (**A4**). Summary plot of the mean AHP amplitude measured in WT and SOD1 motoneurons in the absence (black bars) and presence (gray bars) of oxotremorine (**A5**). (**B)** Representative traces of muscarinic excitatory postsynaptic potentials (musc-EPSPs) induced by different protocols of commissural stimulation (**B1-B2**) in the absence or presence of the muscarinic receptor antagonist, atropine (200 μM, gray trace). Summary plot of the mean musc-EPSP amplitude as a function of the number of shocks and frequency of the commissural stimulation recorded from WT (black bars) and SOD1 (white bars) motoneurons (**B3**). Comparisons between groups were made with two-way ANOVA with Sidak’s multiple comparison tests. Asterisks indicate positive significance (p<0.05), and the numbers in the histogram bars refer to the number of motoneurons recorded from 5 WT mice and 12 SOD1 animals.

In the last part of this study, we addressed the question of whether the alterations reported above in both C-bouton morphology and lamina X ChIns number in L2 segments in early development stages impair the spinal cholinergic neuromodulation of newborn spinal motor networks. For this purpose, we first compared the effects of the broad-spectrum muscarinic receptor agonist, oxotremorine on pharmacologically induced fictive locomotion in *in vitro* spinal cord preparations from P1-P3 WT and SOD1 newborn mice (Fig 7). As recently shown by our group [29], pharmacological stimulation of SOD1 spinal cords with NMA and 5-HT induces WT-like coordinated locomotor activities characterized by left-right and flexor-extensor alternations of motor bursts when recorded extracellularly from the L2 and L5 ventral roots (Fig 7A and 7B). After inducing control fictive locomotion with NMA-5-HT (16 μM each), WT and SOD1 spinal cords were challenged with increasing concentrations of oxotremorine (5, 10 and 20 μM) in the presence of NMA-5-HT (Fig 7A and 7B). Regardless of the mouse genotype, oxotremorine failed to modify the locomotor period at any concentration tested (Fig 7C). In contrast, the amplitude values of the L2 (Fig 7D) and L5 (data not shown) motor bursts were significantly increased in the presence of 5 or 10 μM oxotremorine. Two way-ANOVA showed that this increase in motor burst amplitude was similar between SOD1 and WT littermates. These results indicate that when investigated at the extracellular level, muscarinic receptor activation triggered the same effects in WT and SOD1 spinal motor networks. These data however do not eliminate the possibility of specific modifications in the cholinergic neuromodulation of lumbar Mns. This extracellular analysis was therefore completed by an intracellular investigation of the effects of oxotremorine on motoneuronal membrane properties in spinal cord slices from WT and SOD1 P7-P11 mice (Fig 8). In Mns that were synaptically isolated by adding strychnine (1 μM), gabazine (1 μM) and DNQX (5 μM) to a high cation-containing aCSF, the superfusion of 10 μM oxotremorine induced a depolarization of the membrane potential that was not significantly different between WT and SOD1 Mns (Fig 8A1-2). As previously shown [10] and independent of animal genotype, the oxotremorine-induced depolarization was accompanied by an increase in the input membrane resistance of the Mns (Fig 8A3) and a reduction in the spike after hyperpolarization (AHP, Fig 8A4-5). This latter inhibitory effect was linked to M2 receptor activation as oxotremorine-induced reduction in AHP amplitude was counteracted by the superfusion of the M2 receptor antagonist, AF-DX 116 (50 μM; Fig 8A4). Two way-ANOVA analyses showed that the oxotremorine effects on both input membrane resistance and AHP reduction were similar between SOD1 and WT littermates.

We have previously shown in newborn rat spinal cord slices that electrical stimulations of the ventral commissure induced long-lasting muscarinic excitatory postsynaptic potentials (musc-EPSPs), partly supported by M2 receptor activation in lumbar Mns [26]. These muscarinic responses were dependent on both the commissural stimulation frequency and the number of shocks in the stimulation trains. In WT and SOD1 P7-P11 Mns, we therefore tested the efficiency of different stimulation protocols in inducing musc-EPSPs when applied to the ventral commissure. As in rats, these stimulations triggered long-lasting depolarizations in synaptically isolated Mns (Fig 8B1-2) that were sensitive to the bath-application of the muscarinic receptor antagonist atropine (200 μM, Fig 8B2) and whose amplitudes were dependent on the frequency and number of shocks in the ventral commissure stimulations (Fig 8B3). Two way-ANOVA showed again that musc-EPSP amplitude values were similar when compared between WT and SOD1 Mns.

Altogether these data suggest that the early alterations of the C-bouton system do not impact the cholinergic neuromodulatory influences on newborn Mns in SOD1 spinal networks.

## Discussion

Previous studies have shown that C-boutons contacting motoneurons are subject to morphological changes in physiological and pathophysiological conditions [13,14,17–20]. The present findings confirm and extend this idea by identifying a complex age-related sequence occurring during normal differentiation and maturation of the pre- and post-synaptic partners of C-boutons, a process that appears completely impaired during ALS pathogenesis. Our data also provide, for the first time, evidence that the C-bouton-originating neurons, the lamina X ChIns, degenerate in presymptomatic stages in the SOD1 mouse model. The present study therefore constitutes a major advance in our understanding of spinal motor network physiology and ALS pathophysiology.

### The cholinergic spinal system in WT mice

Rodents are classically considered as adults at P60-75 [30]. Strikingly, in WT mice, we observed that C-boutons undergo long-lasting maturation processes with a progressive increase in area until P40-P75, corroborating the results obtained in rats [31]. In addition, we found that while the C-bouton area and M2 receptor labeling intensity progressively increase from P10 to P75, the postsynaptic membrane portion containing M2 receptors in front of C-boutons progressively decreases. These data therefore suggest that M2 receptors do not invade the postsynaptic territory during the expansion of the presynaptic terminal but rather are clustered in the PSM. At P100, we observed an increase in C-bouton number without changes in their area values and a diffusion of M2 receptors in the PSM of these synapses. As synaptogenesis is classically associated with postsynaptic receptor clustering [32], our data raise the question of whether this sequence of C-bouton enlargement, M2 receptors clustering followed by a renewal of C-terminals on Mns constitutes a cycle that will be repeated all along the animal life span or whether this is an hallmark of developing C-boutons. Few data are available concerning G-protein coupled receptor organization and dynamics in postsynaptic densities. Postsynaptic cluster stability and dynamics are tightly controlled by their anchorage to the postsynaptic actin cytoskeleton [33]. As previously stated, the C-bouton PSM has been recently described as a mosaic domain composed of M2 receptors, Kv2.1 channels and SK2/3 channels clusters [34]. Future studies characterizing the ultrastructure of C-boutons, their associated scaffolding postsynaptic proteins and the role of M2 receptor clusters will be necessary to further assess the physiology of these highly flexible synaptic terminals.

### The cholinergic spinal system in SOD1 mice

Table 2 summarizes the changes we observed across the different parameters analyzed in SOD1 mice compared to age-matched WT animals. We found that as early as birth, the SOD1 spinal cholinergic system markedly differs from the WT with smaller C-boutons and an increased number of lamina X ChIns in L2 segments. During development (P21-P40), SOD1 Mns exhibit a cholinergic hyperinnervation characterized by an increased density of large C-boutons. This synaptic hyperinnervation could be linked to the over-abundance of lamina X ChIns we observed in L2 segments at the same ages. It has been shown that lamina X ChINs in high lumbar segments project to more distal segments [35] and could therefore contribute to the higher C-bouton number observed in SOD1 Mns. In latter stages (up to P75), when Mns have already begun to degenerate, we observed a general deterioration of the SOD1 spinal cholinergic system characterized by a decrease in C-bouton number and area in surviving motoneurons, a decrease of M2 receptor expression in the ventral spinal cord and the degeneration of lamina X ChIns. Interestingly, M2 receptor labeling analysis revealed that the age-related sequence of C-bouton enlargement and M2 receptor clustering described in WT Mns was absent in SOD1 Mns. The distribution of M2 receptors in the PSM juxtaposed to C-boutons then appears altered in SOD1 Mns. Comparable changes in membrane channel localization have been previously reported in Mns. Indeed, a rapid declustering of Kv2.1 has been described in these neurons following peripheral nerve injury [36]. Mounting evidence indicates that dysfunctions of PSM scaffolding proteins are important features of neurodegenerative diseases [37,38]. A proteomic analysis has revealed aberrantly expressed cytoskeletal proteins in the hippocampus of SOD1 mice [39]. The role of these proteins in ALS pathogenesis in spinal motor networks is, however, largely ignored.

**Table 2:** Summary of the changes observed for the different parameters analyzed in SOD1 mice compared to wild-type mice.

	P1	P10	P21	P40	P75	P100
MN perimeter	**-**	**-**	**=**	**=**	**=**	**=**
C-Bouton nb	**na**	**=**	**+**	**+**	**=**	**-**
C-Bouton area	**na**	**-**	**+**	**=**	**=**	**-**
M2 ratio	**=**	**=**	**=**	**=**	**-**	**-**
INT nb in L2	**na**	**+**	**na**	**+**	**na**	**=**

P: postnatal ages.–: smaller, =: equal, +: higher, na: non-available. MN: motoneurone, INT nb in L2: number of Lamina X cholinergic interneurons in L2 segments.

Recent studies have identified specific markers of fast and slow MNs, the matrix metalloproteinase 9 (MMP9) and the estrogen-related receptor β (ERR β), respectively [40,41]. As fast MNs are considered as more vulnerable neurons than slow MNs during ALS pathogenesis, it could have been relevant to investigate whether the cholinergic neuromodulatory system differs between fast and slow MNs during ALS progression. A growing body of evidence now suggests that compensatory mechanisms may occur during ALS pathogenesis leading to a switch of slow type Mns into fast-like phenotype, and vice versa. In the trigeminal motor nucleus of SOD1 mice for example, a subset of slow type MNs exhibits a hypoexcitable shift in rheobase compared to wild type MNs. In contrast, other slow type MNs undergo a hyperexcitable shift in rheobase similar to fast type MNs [42]. In SOD1 mutant rats, in the terminal phase of the disease, slow MNs reinnervate fast muscle fibers and gain some properties of fast motor units [43]. Recently, it has been shown in the SOD1-G85R mouse model that MMP9-immunopositive MNs are present in the spinal ventral horn at 4 months of age while electrophysiological investigation reveals a massive loss of fast type MNs at that stage [44]. This result could be interpreted as the acquisition of a fast-like phenotype by surviving MNs at this time point in the disease but also calls into question the specificity of the MMP9 protein as a selective marker for fast type MNs. Moreover, activity-dependent conversion of fast muscle fibers to slow muscle fibers has been reported in P60 SOD1-G93A mice [16]. This conversion, observed at the effector level itself, is most probably accompanied by phenotypic changes in surviving MNs to increase the motor unit level of activity and to sustain posture and movement.

In addition to these MN type adaptations, neurodegenerative processes appear to be heterogeneous within MN subpopulations in the SOD1 mouse model of ALS. Indeed, *in vivo* imaging of axonal branches has revealed that in individual motor pools, MNs could be divided into two different subpopulations: MNs that exhibit compensatory growth and Mns characterized by axonal dieback [45]. As these data were obtained from motor units innervating muscles with few slow fibers, the authors of this study claimed that fast and slow type MNs could not account for this bimodal distribution but instead revealed differences in MNs vulnerability within a same motor pool [45].

In this context, we think that co-immunostaining experiments with MMP-9 and ERR beta, would not have allowed us to consider that we have specifically discriminated the cholinergic modulation from that of fast MNs to slow MNs throughout ALS pathogenesis.

### Neuronal targets of ALS in the SOD1 spinal cord

The assumption that cortical motor neurons and spinal motoneurons are the only cellular subtypes to degenerate in ALS has often been questioned. A loss of Renshaw cells has been reported in SOD1 spinal networks [46]. The present findings, in addition to corroborating a recent study that showed a decrease in ChAT expression density in lamina X ChIns in the SOD1 mouse [19], further support the fact that motoneurons are not the only neurons to degenerate in the spinal cord of SOD1 mice and that spinal interneurons are also targeted by apoptotic processes in this ALS model. The most rhythmogenic part of the lumbar spinal cord has been shown to be located between the thoracic 12 (Th12) and the L2 segments [47]. Zagoraiou et al, [9] and the present study show a rostro-caudal gradient of lamina X ChIns number in lumbar segments. It has been shown that the genetic silencing of lamina X ChIns does not impact fictive locomotion generation in the *in vitro* spinal cord preparation but alters locomotor task modulation of muscle activity [9]. These results suggest that while lamina X ChIns are abundant in the most rhythmogenic part of the lumbar spinal cord, these neurons are not directly involved in locomotion generation. The present study reveals an abnormal enrichment of lamina X ChIns in L2 segments of SOD1 mice in presymptomatic stages and the degeneration of these L2 ChIns during ALS pathogenesis. We have so far no explanation for this selective regional degeneration. The neuronal and/or non neuronal environment present in this highly excitable part of the spinal cord could, may be, explain this loss of lamina X ChIns during ALS progression.

### Functional impact of C-bouton alterations

Conflicting reports have been made concerning the age-related morphological changes of C-boutons juxtaposed to SOD1 Mns [17–20]. The data presented here also exhibit some discrepancies with these studies that could be linked to differences in the SOD1 model used, the gender of animals used, the method of analysis and the method of labeling C-boutons. Regardless of differences, both previous and the present studies report early alterations in the C-bouton system in SOD1 spinal cord. In the present findings, however, we did not report any functional consequences of these impairments on the spinal cholinergic neuromodulation when assessed at both the extra- and intra-cellular levels in newborn mice. In a recent study, we have shown that the dopaminergic neuromodulation of spinal locomotor networks was similar in WT and SOD1 spinal cord, whereas the dopamine spinal content differed [29]. In the same way, the motor outputs triggered by NMA-5HT bath-applications to the *in vitro* spinal cord preparations from WT and SOD1^G93A^ mice were identical [29] despite previously reported morphological and physiological alterations in the Mns [48,49]. Altogether, these data suggest the existence of compensatory mechanisms to overcome cellular dysfunctions and to maintain homeostasis in SOD1 spinal networks. In P10-P40 animals, the question then arises as to whether the over-abundance of lamina X ChIns in L2 segments we observed and the increased number of C-boutons on SOD1 Mns are part of these compensatory mechanisms or whether this hypercholinergic state is detrimental for Mns and favors pathology onset. C-boutons have been shown to be involved in motor tasks requiring significant Mn discharge such as swimming [9]. In this sense, cholinergic release through C-boutons inhibits AHP expression and increases Mn firing frequency [10]. It has been recently demonstrated that hyperexcitation can be neuroprotective for SOD1 Mns and that the muscarinic receptor antagonist, methoctramine enhanced the accumulation of misfolded SOD1 proteins in these neurons [20]. We could therefore hypothesize that a cholinergic hyperinnervation would constitute a self-defense mechanism in SOD1 Mns. Alterations in M2 receptor clustering could however threaten this potential protective mechanism by modifying acetylcholine efficiency on its receptors. Electrophysiological recordings from mature Mns in slices are still challenging and require very special experimental conditions [50–52]. *In vivo* patch-clamp recordings could be an alternative, but control over the Mn extracellular medium is limited compared to *in vitro* conditions. However, it would be of particular interest to investigate acetylcholine effects on mature WT and SOD1 Mns to further investigate the muscarinic influences received by these neurons and to determine whether this neuromodulatory system is a key contributor to ALS pathogenesis.
